# DCAU-Net: dense convolutional attention U-Net for segmentation of intracranial aneurysm images

**DOI:** 10.1186/s42492-022-00105-4

**Published:** 2022-03-28

**Authors:** Wenwen Yuan, Yanjun Peng, Yanfei Guo, Yande Ren, Qianwen Xue

**Affiliations:** 1 College of Computer Science and Engineering, Shandong University of Science and Technology, Qingdao, 266590 China; 2 The Department of Radiology, the Affiliated Hospital of Qingdao University, Qingdao, 266000 China; 3 Qingdao Maternal & Child Health and Family Planning Service Center, Qingdao, 266034 China

**Keywords:** Deep learning, Intracranial aneurysm segmentation, Magnetic resonance angiography, Multi-scale fusion

## Abstract

Segmentation of intracranial aneurysm images acquired using magnetic resonance angiography (MRA) is essential for medical auxiliary treatments, which can effectively prevent subarachnoid hemorrhages. This paper proposes an image segmentation model based on a dense convolutional attention U-Net, which fuses deep and rich semantic information with shallow-detail information for adaptive and accurate segmentation of MRA-acquired aneurysm images with large size differences. The U-Net model serves as a backbone, combining dense block and convolution block attention module (CBAM). The dense block is composed of a batch normalization layer, an randomly rectified linear unit activation function, and a convolutional layer, for mitigation of vanishing gradients, for multiplexing of aneurysm features, and for improving the network training efficiency. The CBAM is composed of a channel attention module and a spatial attention module, improving the segmentation performance of feature discrimination and enhancing the acquisition of key feature information. Owing to the large variation of aneurysm sizes, multi-scale fusion is performed during up-sampling, for adaptive segmentation of MRA-acquired aneurysm images. The model was tested on the MICCAI 2020 ADAM dataset, and its generalizability was validated on the clinical aneurysm dataset (aneurysm sizes: < 3 mm, 3–7 mm, and > 7 mm) supplied by the Affiliated Hospital of Qingdao University. A good clinical application segmentation performance was demonstrated.

## Introduction

Intracranial aneurysms are prevalent at 3% in the adult population [1]. Aneurysmal protrusions are formed owing to abnormal enlargements of cerebral arterial vessels. A ruptured aneurysm can cause a subarachnoid hemorrhage (SAH), with a potentially lethal outcome. SAH survivors often suffer from long-term cognitive impairments. Therefore, early detection and evaluation of intracranial aneurysms are important for timely clinical examination that enables early detection of aneurysms, in turn enabling appropriate rupture-prevention strategies. Magnetic resonance angiography (MRA), which enables assess to very small (2–3 mm) lesion areas for clearer visualization of aneurysms, is important for aneurysm screening [2]. MRA is not associated with radiation exposure, is highly sensitive, and emphasizes vasculature. Therefore, it is the preferred technique for screening intracranial abnormalities in asymptomatic patients, and was used in the present study. Specifically, the intracranial aneurysm images in this study were obtained using three-dimensional (3D) time of flight (TOF) MRA.

With the increasing use of deep learning in medical applications, convolutional neural networks (CNNs) have been repeatedly shown to perform well on segmentation of medical images, and have become gradually accepted as a solution for improving diagnostic accuracy and clinical decision making [3–5]. With respect to the cerebral artery images, extraction and segmentation of the regions of interest is key for diagnosis of intracranial aneurysms. Accurate segmentation facilitates geometric quantification and evaluation of the rupture risk of intracranial aneurysms. Therefore, deep learning-based methods can be used for building appropriate CNNs for automatic segmentation of aneurysm-containing regions, enabling more accurate approaches to treating aneurysms. This study makes the following contributions to the aneurysm segmentation problem:

1. 3D aneurysm-containing MRA-acquired images have richer contextual information; thus, a 3D network is designed and trained for adaptive segmentation of aneurysm-containing regions.

(2) The multi-scale feature fusion block fuses the feature information of different layers, merging the semantic information of higher layers with the edge information of lower layers, which improves the method’s segmentation performance on aneurysms.

(3) The designed DCAU-Net was validated on the clinical data provided by the Affiliated Hospital of Qingdao University, and good segmentation results were achieved.

### Machine learning-based methods

Many methods have been proposed for medical image segmentation. Examples include the watershed algorithm [6], the lattice Boltzmann method [7], genetic algorithms [8], and the 3D Otsu threshold segmentation algorithm [9]. Machine learning-based aneurysm segmentation methods focus on segmenting the cerebrovascular structure images and then applying this information to the segmentation of intracranial aneurysm images. For example, segmentation of intracranial aneurysm images acquired using 3D Rotational Angiography and computed tomography angiography (CTA) based on a geometric deformation model [10], a geodesic active contour (GAC) combined with Euler’s elastic model for segmenting CTA-acquired large aneurysm images (size, > 25 mm) [11], and automatic segmentation of aneurysm images using the improved threshold-based level set method [12] have been proposed.

### Deep learning-based methods

Recently, CNN-based methods [13] have been shown as very effective for feature extraction. The network structure is constantly being revised and improved, and has evolved from the original LeNet [14], to VGGNet [15], to GoogleNet [16], to ResNet [17], and to DenseNet [18]. The fully convolutional network (FCN) [19] is a pioneering structure in the field of image segmentation; the FCN amounts to the first proposed end-to-end pixel-to-pixel network for semantic segmentation. The FCN does not limit the size of the input image and combines local feature information with semantic features using a jump structure. The segmentation performance of this network is significantly better than that of other networks; however, the network is not sensitive to the image details. The U-Net [20] architecture draws on the FCN. The U-Net is an end-to-end symmetric network that includes a contraction path (for feature acquisition) and an expansion path (for precise positioning). It can freely deepen the network structure according to its own dataset and is widely used in the field of medical segmentation; its segmentation performance is very good [21–23].

The main existing methods are based on the maximal intensity projection (MIP) algorithm [24], which projects 3D images onto two-dimensional (2D) images in different directions according to the voxel intensity, following which a 2D CNN is constructed for feature detection [25, 26]. Jin et al. [27] introduced a bidirectional convolutional long short-term memory module based on the U-Net architecture, for learning the spatial and temporal information of aneurysms in different 2D digital subtraction angiography (DSA) sequences for end-to-end training. The system has some limitations, and it remains difficult to detect and segment small aneurysms. Compared with 2D CNNs, a 3D CNN [28] emphasizes the spatial structure information of images and performs better. Park et al. [29] developed the HeadXNet model as a neural network segmentation model, for accurately predicting intracranial aneurysms from head CTA images; although the method’s prediction performance on aneurysms is very good, it has some limitations – the primary one is that the method is not generalizable to different scanners and imaging protocols. Recently, Shi et al. [30] proposed DAResUNet, which combines residual blocks and dual attention blocks [31] for analysis of CTA-acquired aneurysm images. The model was trained and validated on a large volume of clinical data. Of the predicted negative cases, 99% were highly trusted and could be used for reducing the clinicians’ workload. The research focused only on CTA-acquired aneurysm images. Shahzad et al. [32] proposed a double-path CNN architecture for capturing contextual information at different resolutions, achieving a sensitivity close to 100% for aneurysms larger than 100 mm, on the atherosclerosis cohort. However, deep learning-based models (DLMs) can only evaluate images scanned on iCT machines; the segmentation performance on CTA images acquired using different scanners is unknown and requires further validation. In ref. [33], a deep learning-based method, GLIA-Net, was proposed without redundant preprocessing and was applied directly to CTA-acquired images. The model consisted of a global position network and a local segmentation network, avoiding the loss of global information owing to cropped image blocks. The entire 3D CTA image, that is, the global map, was used as input to the image-processing network, for capturing the global position information of the current block; the slices in the global image were used as local images, for correctly capturing small target regions. Yet, the model was not validated on other types of images. In ref. [34], Ma and Nie used the 3D nn-UNet model [35] for training and evaluating the performance on the MICCAI 2020 CADA dataset, and achieved good segmentation results. It is worth mentioning that this method required significant amount of hardware and was time-consuming. In ref. [36], “weak labels” were proposed instead of manual voxel labeling, to reduce the labeling time, following which a customized U-Net network was trained for segmentation of the different parts of TOF MRA-acquired intracranial aneurysm images. This method did not use a novel model method and focused on pre-image processing. Çiçek et al. [37] proposed a 3D U-Net for three-dimensional biological images.

The above methods focus on the segmentation of CTA-acquired aneurysm images; at the same time, methods for segmenting MRA-acquired images are lacking. Therefore, the purpose of this study was to develop a CNN for adaptive segmentation of MRA-acquired intracranial aneurysm images. The proposed network combines dense blocks and a CBAM [38]. The network is taught to recognize target features and apply them to the segmentation of intracranial aneurysm images. The multiscale fusion block (MFB) in the up-sampling part fuses contextual information at different scales, which is helpful for improving the segmentation accuracy of aneurysm-containing images. In addition, 3D images have richer spatial information, yet there are only a few studies on the automatic segmentation of intracranial aneurysm images from 3D TOF MRA imaging data. Therefore, the purpose of this study was to investigate the clinical potential of deep learning for automatic segmentation of intracranial aneurysm images from 3D TOF MRA imaging data.

### Methods

#### Pre-processing

In this step, we mainly performed the following operations: image cropping, z-score standardization, and data enhancement. These preprocessing operations extracted 3D images containing aneurysm regions, making the data more conducive to network training.

**Fig. 1:**
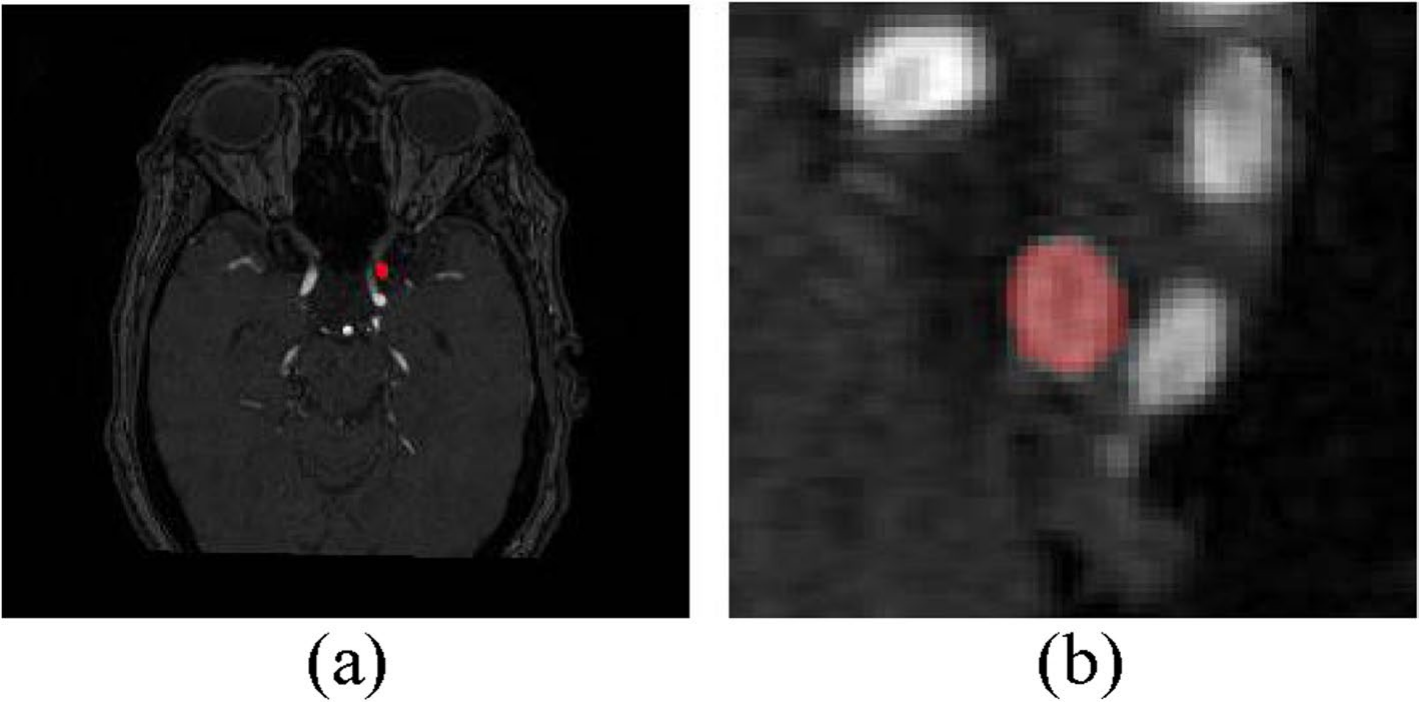
The intracranial aneurysm image and its ground truth. **a** Original 3D TOF MRA image of an intracranial aneurysm in the sagittal view, with the red marker indicating the aneurysm-containing region; **b** Image of the aneurysm after pre-processing

In the image cropping operation, the pixel ratio of the target region was too small, and cropping off the zero-pixel region still suffered from the foreground–background imbalance. In addition, the sizes of the original aneurysm-containing images were inconsistent, with the smallest image of 512 × 512 × 64 and the largest image of 1024 × 1024 × 160. With original 3D images as the input to the model, large non-target regions would not be efficient memory- and computation-wise, which is not conducive to the segmentation accuracy of aneurysms. Therefore, the aneurysm location was set as the image center, and the images were cropped to 64 × 64 × 64 uniformly, for reducing the foreground–background ratio, as shown in Fig. 1.

Gray normalization is a necessary preprocessing step for image segmentation. Gray normalization enables efficient feature extraction and speeds up the gradient descent for finding the optimal solution. We chose the *z*-score standardization method [39], which normalizes features to the same scale, which is convenient for comprehensive feature comparison and weighting. The *z*-score standardized image data conformed to the normal distribution (mean, 0; standard deviation, 1); the *z*-score was obtained from the data and the distribution parameters as follows:

1 $$z = \frac{x - \mu }{\sigma }$$

where *x* represents the pixel-level MRA-acquired sequence data, *μ* is the mean value of the pixel-level MRA-acquired sequence data, and *σ* is the standard deviation of the pixel-level MRA-acquired sequence data. Because the amount of available data is typically relatively small, data enhancement operations are often performed for preventing overfitting during model training [40]. We used random flips and random strength enhancement for expanding our dataset, which improved the model’s generalizability. We added an offset of 0.1 to each channel of the preprocessed intracranial aneurysm images, and applied a random mirror flip with a probability of 0.5, to the *x*, *y*, and *z* axes data.

### Proposed model

#### Network structure

**Fig. 2:**
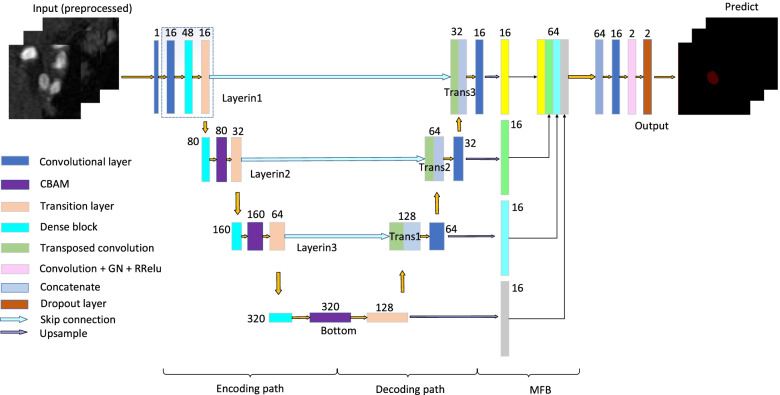
Schematic of the proposed network (DCAU-Net). The framework is shown in the 2D form, for the sake of convenient viewing

The study used DCAU-Net, corresponding to an improved U-Net. The network consisted of three parts: (1) a down-sampling (encoding) path, (2) an up-sampling (decoding) path, and (3) a MFB. The specific structures are shown in Fig. 2. The network was an end-to-end FCN. The input to each layer of DCAU-Net was $b \times c \times h \times w \times d$, where *b* represents the batch size, *c* represents the number of channels, while *h*, *w* and *d* represent the three dimensions of the input image.

The four convolution blocks contain Layerin1–Layerin3, and Bottom. Layerin1 consisted of a conventional convolution block, a dense convolution block, and a transition layer. The conventional convolution block consisted of two convolution layers (kernel size, 3 × 3 × 3; stride size, 1 × 1 × 1), where group normalization (GN) [41] was used instead of batch normalization (BN) [42] for reducing the memory demand. The remaining blocks contained a two-layer dense convolutional block and a CBAM, followed by a transition layer. The CBAM was placed before the pooling layer, to suppress unimportant features and further focus on the aneurysmal feature regions. The transition layer mainly consisted of a BN layer, a randomly rectified linear unit (RReLU) function, a convolution layer with a 1 × 1 × 1 convolution kernel, and an average pooling layer. Among these, the 1 × 1 × 1 convolutional layer was used for reducing the number of channels. The average pooling layer (convolutional kernel, 2 × 2 × 2; step size, 2 × 2 × 2) allowed to reduce the number of the network parameters, minimize overfitting, and reduce the model’s complexity. On the other hand, the transition layer solved the problem of changing the number of channels brought by multiple dense blocks in series, unified the size of the feature maps of each layer, and facilitated the skip connection during the up-sampling. The transition layer reduced the image to a half of the original image each time, reducing the dimensions of the feature map from 64 × 64 × 64 to 4 × 4 × 4.

In the up-sampling path, a transposed convolutional layer with a filter size of 3 × 3 × 3 and stride size of 2 × 2 × 2 was first applied to each block. The down-sampled feature maps were fused to the up-sampled feature maps by the skip connection, to ensure that the sizes of the feature map matched during the up- and down-sampling. The image dimensions increased from 4 × 4 × 4 to 64 × 64 × 64. In this study, the transposed convolution and the corresponding down-sampling layer were fused to the feature maps (Trans1–Trans3). This alleviated the vanishing gradient problem and increased the training speed. For the up-sampling path, two conventional convolutional blocks were applied at the end of each layer, reducing the number of feature maps after the fusion of the transposed convolution and down-sampling paths. We set padding to 1, for maintaining the output dimensions of all the convolutional layers in the encoding and decoding paths.

The MFB fused the context information of different scales during the up-sampling. Each layer was size-unified by the trilinear interpolation of the Upsample function, following which feature fusion was performed. This compensated for the lack of high-level detail information and little semantic information at the lower levels during the up-sampling of the U-Net network. The introduction of the CBAM allowed to further learn the distinguishing feature information of aneurysms, from both the channel and spatial dimensions. The number of channels for the foreground and background segmentation was then reduced to two, using a convolution layer with a filter of 3 × 3 × 3 and a step size of 1 × 1 × 1. Finally, we added a dropout layer, to prevent overfitting.

### Dense block

**Fig. 3:**
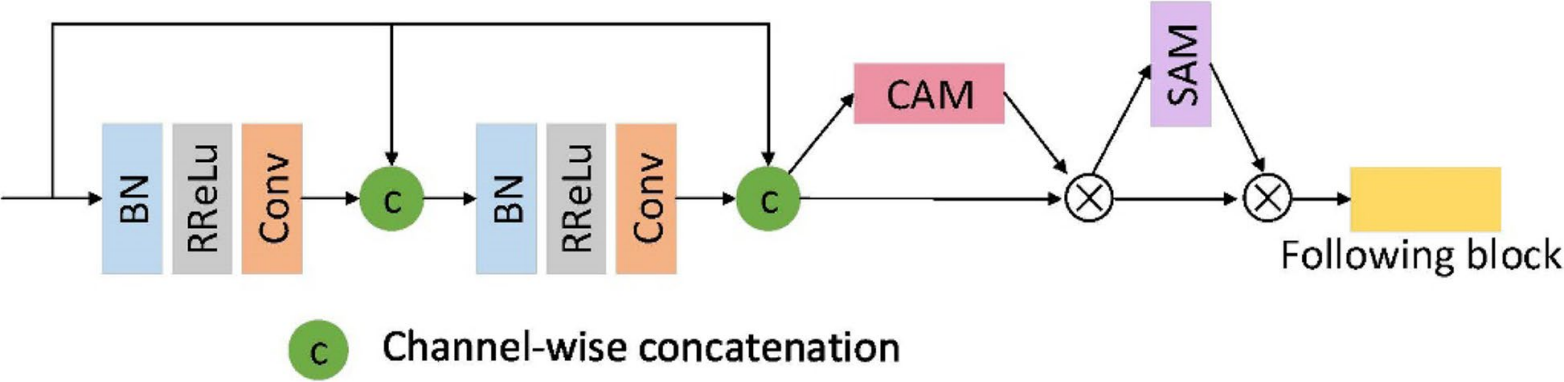
Integration of the CBAM with the dense block

In this study, a dense connection module in DenseNet was used for improving the information transfer between different layers. Figure 3 shows the specific connection structure of the dense block and the CBAM. The dense block used a pre-activation mode, consisting of the BN–RReLu–Conv sequence. Using BN layers earlier in pre-activation can improve a model’s regularization ability. Each densely connected block contained two 3 × 3 × 3 convolutional layers and two feature fusions. After the feature map of the input densely connected block underwent the convolution operation, the generated feature map was fused with the original feature map, forming a new feature map. Then, it was presented to the channel attention module (CAM), for obtaining weighted results, following which it was presented to the spatial attention module (SAM). Finally, the weighted features were presented to the next convolution block.

### The CBAM

The CBAM combined two attention mechanisms of channel and space and multiplied the attention feature map with the input feature map, allowing the network to learn the most distinguished features in data. Section 4 specifically discusses the impact of different CBAM module arrangements on the segmentation performance.

**Fig. 4:**
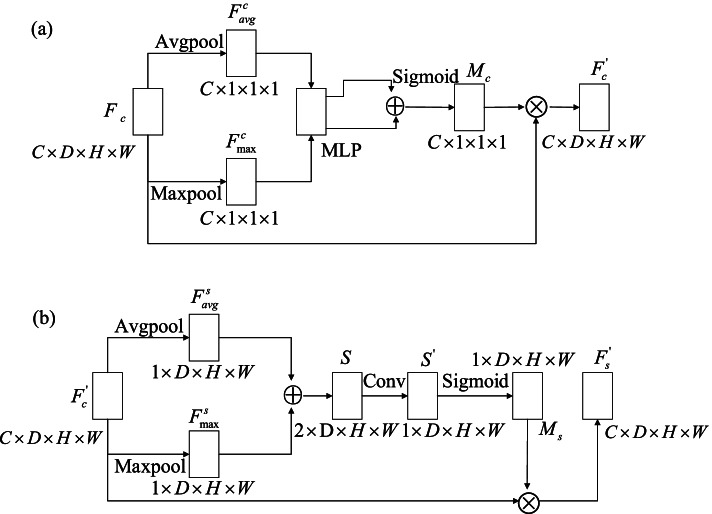
The complete CBAM module. **a** Structure diagram of channel attention module; **b** Structure diagram of spatial attention module

The channel attention sub-module, which utilizes the channel relationship between features, is shown in Fig. 4(a). The workflow is as follows:

(1) The feature map $F_{c}$ uses global average pooling and global maximum pooling for obtaining two spatial context descriptors: $F_{avg}^{c}$ and $F_{\max }^{c}$. The feature dimensions change from $C \times D \times H \times W$ to $C \times 1 \times 1 \times 1$.

(2) $F_{avg}^{c}$ and $F_{\max }^{c}$ are presented to a shared multilayer perceptron (MLP), and then the output features perform concatenation operations. The spatial feature map $M_{c}$ is obtained through the sigmoid activation. The dimensions of the feature map are $C \times 1 \times 1 \times 1$. The mathematical expression for $M_{c}$ is

2 $$\begin{gathered} M_{c} \left( F \right) \\ = \sigma \left( {MLP\left( {AvgPool\left( F \right)} \right) + MLP\left( {MaxPool\left( F \right)} \right)} \right) \\ = \sigma \left( {W_{1} \left( {W_{0} \left( {F_{avg}^{c} } \right)} \right) + W_{1} \left( {W_{0} \left( {F_{\max }^{c} } \right)} \right)} \right) \\ \end{gathered}$$

where σ represents the sigmoid function. Note that the MLP weights, $W_{0}$ and $W_{1}$, are shared for both inputs, and the rectified linear unit (ReLU) activation function is followed by $W_{0}$.

(3) $M_{c}$ is multiplied by the initial input feature map $F_{c}$ to obtain $F_{c}^{^{\prime}}$, which is the output of the CAM.

The detailed structure of the spatial relationship between the features of the spatial attention submodules is shown in Fig. 4(b), and is explained as follows:

1. The feature map $F_{c}^{^{\prime}}$ from the output of the CAM is used as the input to the SAM; then, global average pooling and global maximum pooling are performed for generating two maps, $F_{avg}^{s}$ and $F_{\max }^{s}$. The dimensions of the feature map change from $C \times D \times H \times W$ to $1 \times D \times H \times W$.

(2) $F_{avg}^{s}$ and $F_{\max }^{s}$ are concatenated, for obtaining S with the dimensions of $2 \times D \times H \times W$. Then, by the 7 × 7 × 7 convolution, the dimensionality is reduced to a channel number for obtaining $S^{^{\prime}}$, that is, $1 \times D \times H \times W$. The dimensions of the feature map are $1 \times D \times H \times W$.

(3) $S^{^{\prime}}$ uses the sigmoid activation function for obtaining $M_{s}$. Its dimensions are $1 \times D \times H \times W$. It is defined as

3 $$\begin{gathered} M_{s} \left( F \right) \hfill \\ = \sigma \left( {f^{7 \times 7 \times 7} \left( {\left[ {AvgPool\left( F \right);MaxPool\left( F \right)} \right]} \right)} \right) \hfill \\ = \sigma \left( {f^{7 \times 7 \times 7} \left( {\left[ {F_{avg}^{s} ;F_{\max }^{s} } \right]} \right)} \right) \hfill \\ \end{gathered}$$

where $f^{7 \times 7 \times 7}$ represents the convolution operation with a convolution kernel of 7 × 7 × 7, $F_{avg}^{s}$ represents the average pooling feature through the channel, and $F_{\max }^{s}$ represents the maximum pooling characteristic through the channel.

### Normalization

The normalization process ensures that the data distributions for each round of training are consistent. Therefore, in the present work normalization was performed after each convolutional layer, that is, layer-processed data were normalized to the standard distribution (mean, 0; standard deviation, 1). This standardization improved convergence. The proposed network used two normalization layers: (1) 3D BN and (2) 3D GN.

For the convolutional layer in the dense block, 3D BN was used, defined as

4 $$y = \gamma^{\left( k \right)} \times \frac{{x^{\left( k \right)} - E\left[ {x^{\left( k \right)} } \right]}}{{\sqrt {Var\left[ {x^{\left( k \right)} } \right]} }} + \beta^{\left( k \right)}$$

where $x^{\left( k \right)}$ is a 3D input vector, $E\left[ {x^{\left( k \right)} } \right]$ is the average for each neuron in a batch of 3D data, and $\sqrt {Var\left[ {x^{\left( k \right)} } \right]}$ is the standard deviation for the *k*-th neuron. The parameters γ and *β* are learnable parameters, representing the scale and deviation, respectively.

The dimensions of the proposed network were $\left[ {B,C,H,W,D} \right]$, with each neuron corresponding to a channel. We calculated the mean and variance of the $B \times H \times W \times D$ values and then performed a reconstruction transformation. The BN layer improved and stabilized the network’s training process, and alleviated the vanishing gradient problem. However, the dataset that was used in this study consisted of 3D images, and the input feature space was too large; thus, for training the network the batch size was set to eight. Note that with smaller batches the batch mean and variance cannot be estimated correctly.

To stabilize the data mean and variance estimation accuracy during the training process, a group convolution layer in the traditional convolution block was used for data normalization. The normalization was performed as follows:

5 $$y = \gamma \times \frac{1}{{\sigma_{i} }}\left( {x_{i} - \mu_{i} } \right) + \beta$$

6 $$\mu_{i} = \frac{1}{m}\sum\limits_{{k \in S_{i} }} {x_{k} } ,\sigma_{i} = \sqrt {\frac{1}{m}\sum\limits_{{k \in S_{i} }} {\left( {x_{k} - \mu_{i} } \right)^{2} + \varepsilon } }$$

where $i = \left( {batchsize,iN,iC,iD,iH,iW} \right)$ indexes a five-dimensional vector of a 3D image, $x$ is the feature of a layer, $\mu$ and σ represent the mean and the standard deviation, respectively; $\varepsilon$ is a small constant; $S_{i}$ is a set of pixels for calculating the mean and the standard deviation, and $m$ is the size of the set, which determines the normalization method.

Because the number of output channels for each traditional convolutional layer is $2^{i}$, the feature channels C through the group convolutional layer were divided into four groups. Each group contained $2^{i} /4$ feature channels. The mean and the variance of $\left( {H,W,D} \right)$ were computed according to the four groups. This improved the expressive ability of the network and made the network more flexible with respect to learning the features of the distribution between the different groups. To demonstrate that the GN layer improves the segmentation accuracy of intracranial aneurysms, an ablation experiment was performed using the ADAM dataset.

### Activation function

The activation function introduces nonlinearity into neural networks, which improves their generalizability and enables treating nonlinearly separable problems. The ReLU [43] function is among the most widely used activation functions, and can improve the network’s convergence. The ReLU function is analytically defined as follows:

7 $$f_{{{\text{Re}} lu}} = \max \left( {0,x} \right)$$

Note that the ReLU function forces the output of the *x* < 0 region to zero, which masks features in this region. This may cause the model to fail to effectively learn aneurysm-related features. To overcome this, the RReLU function was used for adding a linear term (for treating negative inputs). Thus

8 $$y = \left\{ {\begin{array}{*{20}c} {x,} & {x \ge 0;} \\ {ax,} & {x < 0.} \\ \end{array} } \right.$$

where $a \sim U\left( {l,u} \right),l < u$ and $l,u \in \left[ {0,1} \right)$. The value of slope $a$ was drawn from a uniform distribution $U\left( {l,u} \right)$. Ablation experimentlists the effectiveness of the RReLU activation function with respect to improving the segmentation performance on intracranial aneurysms in Section 4.

### Loss function

An intracranial aneurysm typically accounts for a small proportion of an image that contains it, and improper use of the loss function will cause class imbalance problems. The cross-entropy loss function was used for testing the extent of the similarity between the predicted results and those obtained using the manual segmentation mask. It is easy to make the loss reach a local minimum, which causes the model to focus on the background region during training. It is difficult to accurately predict the lesion region. The Dice loss function [44] is suitable for solving class imbalance problems, and it is defined as follows:

9 $$L_{dice} = 1 - \frac{{2\sum\limits_{x = 1}^{N} {p\left( x \right)q\left( x \right)} }}{{\sum\limits_{x = 1}^{N} {p\left( x \right)} + \sum\limits_{x = 1}^{N} {q\left( x \right) + e} }}$$

where $N$ is the number of categories (label classes), $p\left( x \right)$ is the model predicted value, $q\left( x \right)$ is the label value from manual segmentation, and $e$ is a small smoothing constant, to prevent the denominator from becoming zero and the gradient from disappearing.

The convergence speed of the Dice loss function becomes lower during the later stages of training. During the learning process, instabilities can be easily incurred owing to the high data variance, making it difficult to improve the segmentation accuracy of the method. Therefore, a weighted combination of the Dice and entropy loss functions was used in the present study, and the corresponding expression was

10 $$L = \left( {1 - \alpha } \right)L_{dice} + \alpha \left( { - \sum\limits_{x = 1}^{N} {p\left( x \right)\log \left( {q\left( x \right)} \right)} } \right)$$

where $\alpha$ is the fractional weight (on the 0–1 scale) for balancing the Dice and cross-entropy loss function contributions. To balance the background and target region pixels, we set $1 - \alpha$ and $\alpha$ to 0.3 and 0.7, respectively, during our experiments.

### Experiment

#### Dataset

Two different datasets were used for testing the generalizability of the DCAU-Net network with respect to the segmentation of intracranial aneurysm-containing regions. One dataset was from the competition MICCAI 2020 ADAM (http://adam.isi.uu.nl/data). The training set is publicly available and includes 113 cases. Among these, data for 93 patients presented at least one untreated, unruptured intracranial aneurysm. The other dataset was provided by the Department of Radiology, the Affiliated Hospital of Qingdao University. The data in this dataset were collected using Philips 1.5 TMR, Siemens 3.0 TMR, GE Signa 3.0 TMR, and GE 1.5 TMR vascular imaging systems. The images were in the DICOM format, with the image resolution of 512 × 512 × 140 (the *z*-axis was not uniform). These 3D TOF-MRA image data were manually annotated by two experienced radiologists and double-checked, with consistent results as the diagnostic criteria for aneurysms. This dataset contained 3D TOF MRA images of 376 patients, collected from January 2012 to December 2019. Table 1 lists the detailed information about the dataset.

**Table 1:** Characteristics of the 376 clinical data samples provided by the Department of Radiology, the Affiliated Hospital of Qingdao University

Size (mm)	< 3	3–7	> 7
Gender			
Male	49	59	52
Female	42	151	23
Age (year)			
30–60	28	80	21
> 60	63	130	54
Number of aneurysms			
1	85	200	67
2	6	10	7
3	0	0	1

The images were uniformly converted into the NII format for preprocessing. For these two datasets, 3D TOF MRA images were selected as the input, and the ground truth labels were slightly different. The segmentation mark annotation of the MICCAI 2020 ADAM included three parts: background (labeled 0), untreated and unruptured aneurysms (labeled 1), and treated aneurysms (labeled 2). The clinical dataset used only two labels: background (labeled 0) and untreated, unruptured aneurysms (labeled 1). The goal was to automatically segment the untreated and unruptured aneurysms. Therefore, label 2 of the MICCAI 2020 ADAM dataset was ignored during the evaluation and was not considered.

### Experimental configuration

Table 2 lists the hyperparameter values of the network. These hyperparameters were obtained from the validation set. The intracranial aneurysm segmentation problem was treated as a two-class classification problem. Only the untreated and unruptured aneurysm areas were regarded as positive, and the other parts were regarded as negative, allowing to more accurately segment the untreated aneurysm parts from the 3D TOF MRA-acquired aneurysm-containing images.

**Table 2:** Parameter values of the proposed model

Layer	Filter	Kernel	Stride	Input
Layerin1	16	3 × 3 × 3	2	1 × 64 × 64 × 64
Layerin2	32	3 × 3 × 3	2	16 × 32 × 32 × 32
Layerin3	64	3 × 3 × 3	2	32 × 16 × 16 × 16
Bottom	128	3 × 3 × 3	2	64 × 8 × 8 × 8
Trans1	64	3 × 3 × 3	2	128 × 4 × 4 × 4
Trans2	32	3 × 3 × 3	2	64 × 8 × 8 × 8
Trans3	16	3 × 3 × 3	2	32 × 16 × 16 × 16
MFB	64	-	-	16 × 64 × 64 × 64
CBAM	64	-	-	64 × 64 × 64 × 64
Output	2	3 × 3 × 3	1	16 × 64 × 64 × 64

The ADAM dataset was randomly partitioned into a training set (*n* = 70), a validation set (*n* = 20), and a test set (*n* = 23). For the clinical dataset of the Affiliated Hospital of Qingdao University, the model’s adaptability and generalizability were tested according to three aneurysm sizes.

The analysis code was written in Python, and the network architecture was based off the PyTorch framework. The model was trained and tested on GPU GeForce RTX 2080Ti, using two graphics processing units each time. During the model training, the Adam algorithm was used for parametric optimization. The initial learning rate was 0.001, and the ReduceLROnPlateau function was used for dynamically adjusting the learning rate. When the evaluation index did not improve for 10 consecutive rounds, the learning rate was reduced by 0.1. Overall, 300 rounds of training were performed, and the batch size of each round was 8.

Filter represents the number of channels in each layer, kernel represents the kernel size of the convolutional layer. Stride represents the step size of the average pooling layer in down-sampling, and represents the step size of the transposed convolutional layer in up-sampling. The output of the last layer is 2 × 64 × 64 × 64.

To evaluate the performance of the proposed method, four classical segmentation performance indicators were used: (1) the Dice metric, (2) sensitivity, (3) specificity, and (4) precision. Among these, the Dice metric is used for computing the similarity between predicted and ground truth values, with the metric value in the [0,1] range; the higher the value, the better the segmentation quality.

11 $$Dice = \frac{2TP}{{FP + 2TP + FN}}$$

In the above, *TP*, *FP*, and *FN* represent the number of true positive, false positive, and false negative calls, respectively.

Sensitivity is the probability of a condition-positive patient to be correctly diagnosed as positive; this metric is known as the true positive rate, or the recall rate. The higher the sensitivity, the more likely are aneurysm-containing regions to be categorized as such.

12 $$Sensitivity = \frac{TP}{{TP + FN}}$$

Specificity refers to the probability of a negative call for a truly condition-negative patient; it is also known as the true negative rate.

13 $$Specificity = \frac{TN}{{FP + TN}}$$

where *TN* represents the number of true negative calls. The higher the specificity, the more likely are healthy-tissue regions to be correctly categorized as such.

Precision is the fraction of correctly predicted positive cases (aneurysm-containing regions) to all positive calls (corresponding to the sum of true- and false-positive calls).

14 $$Precision = \frac{TP}{{TP + FP}}$$

## Results and discussion

**Fig. 5:**
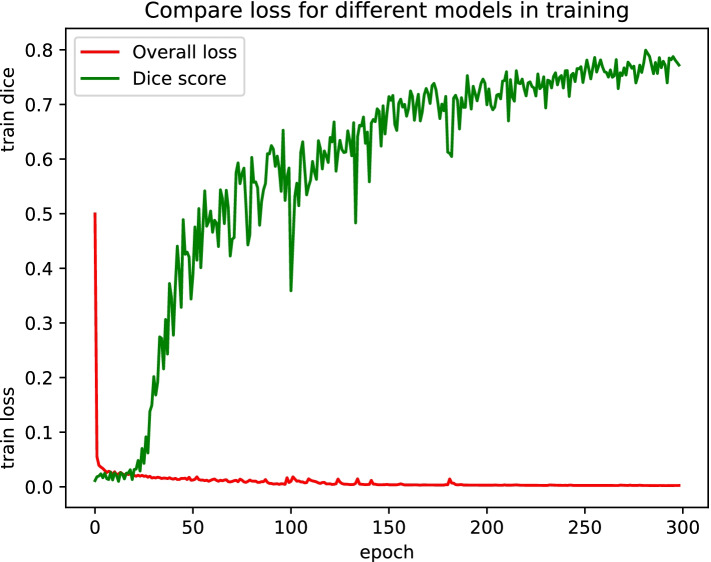
Evolution of the overall loss functions and dice score during the 300 rounds of training, for the MICCAI 2020 ADAM dataset

**Fig. 6:**
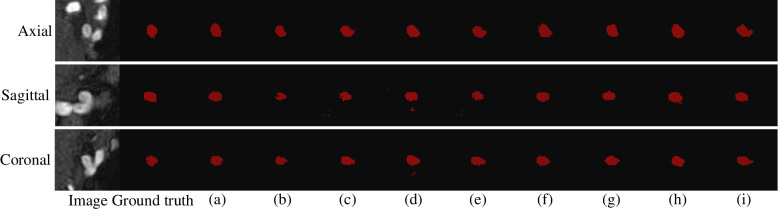
Prediction maps for the models with different components, on the MICCAI 2020 ADAM testing set. The first column is the MRA image of an aneurysm, while the second column shows the manually obtained marking mask. **a **The proposed model; **b **U-Net; **c **U-Net with the dense block; **d **U-Net + CAM; **e **U-Net + SAM; **f **U-Net + CBAM; **g **U-Net + dense block + CBAM; **h **BN only; **i **With ReLU activation

Experiments were conducted for validating the effects of the above-listed key components. For the MICCAI 2020 ADAM dataset, ablation experiments were performed using the training, validation, and testing subsets; the final segmentation results were evaluated, and are listed in Table 3. Figure 5 shows the overall loss function values and Dice score during the 300 training round of DCAU-Net. The combination of the cross-entropy and Dice loss functions that was used for the model training ensured faster convergence toward the minimal loss configuration, proving that the proposed model has a high learning efficiency during training and is suitable for solving the segmentation problem of intracranial aneurysms. The 3D network model used in this study had to be presented with aneurysm images in three dimensions (axial, sagittal, and coronal). Owing to the small sizes of aneurysms, the network was presented with 64 × 64 × 64 MRA-acquired aneurysm-centered images. The different methods are compared in Fig. 6. The segmentation prediction results for example aneurysms suggest that the DCAU-Net network segments the aneurysms well, with the segmentation results closer to those for the manual segmentation mask.

**Table 3:** Comparison of the performances of key components on the MICCAI 2020 ADAM testing set

Component	Dice	Sensitivity	Specificity
Ours	0.7455	0.7881	0.9996
UNet	0.4629	0.6077	0.9992
UNet + Dense	0.5424	0.6068	0.9995
UNet + CAM	0.6783	0.6254	0.9998
UNet + SAM	0.6426	0.7850	0.9997
UNet + CBAM	0.6813	0.7398	0.9997
UNet + Dense + CBAM	0.7292	0.7378	0.9996
Use BN	0.6612	0.6829	0.9996
Use ReLU activation	0.5731	0.6560	0.9993

The results of these experiments show that the Dice value for DCAU-Net reached 74.55% on the testing set. The basic U-Net network exhibited the lowest Dice of 46.29%, which is not suitable for segmenting aneurysm-containing images. The Dice value for U-Net with dense blocks was 7.95% higher compared with that for U-Net alone, proving the effectiveness of dense connections. The combination of U-Net and CBAM was less sensitive than the system that used only the SAM, but the Dice value was higher than that for U-Net combined with a single spatial or channel attention, indicating the effectiveness of the CBAM for segmenting aneurysm-containing regions. To validate the effectiveness of the MFB, a multi-scale fusion block was added to the improved U-Net up-sampling part. The Dice value increased by 1.63%, and the sensitivity increased by 5.03%, indicating that the MFB effectively improved the segmentation performance on aneurysm-containing images. The Dice value and the sensitivity of the traditional convolution block using the BN layer were, respectively, 8.43% and 10.52% lower than those of the currently proposed algorithm. This shows that the GN layer effectively improved the segmentation accuracy for aneurysm-containing images while reducing the memory space. The Dice value obtained using the RReLU function was 17.24% higher than that obtained using the ReLU function, proving the effectiveness of the RReLU function.

**Fig. 7:**
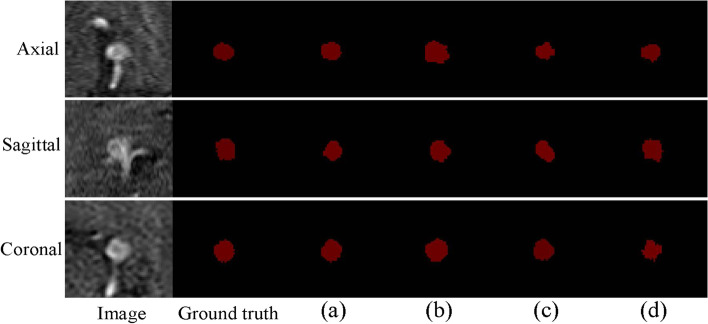
Prediction maps of different attention modules, on the ADAM test set. **a **The proposed model; **b **SENet; **c **SKNet; **d **BAMNet

**Fig. 8:**
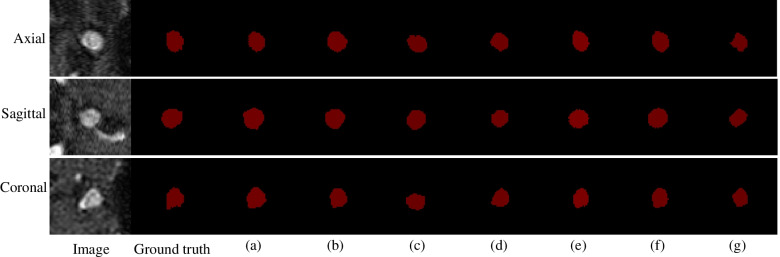
Prediction maps of different models, on the ADAM test set. **a **The proposed model; **b** MIP + 2D CNN; **c** HeadXNet; **d** DeepMedic; **e** GLIANet; **f** DAResUNet; **g** 3D U-Net

To demonstrate that the CBAM can improve the segmentation accuracy of intracranial aneurysm-containing images, several classical attention modules [45–47] were used for comparison experiments. The segmentation results in Table 4 show that the CBAM’s ability to learn aneurysm-related features was better than that of the other considered attention modules. The proposed network performed well on segmenting the entire region and the edge of the aneurysm, as shown in Fig. 7. This shows that the CBAM is more suitable for segmenting images containing intracranial aneurysms. We also conducted comparative experiments against other advanced models, using the ADAM dataset. In Table 5, the average Dice, precision, and sensitivity values, obtained using the DCAU-Net method are listed, exhibiting the highest values. Compared with BAMNet [47] and with GLIA-Net [33], the model in this study exhibited little difference in terms of the average Dice value, but there were obvious differences in terms of the average sensitivity and precision. The average sensitivity and precision for the model in this study were 7.82% and 2.88% higher than the corresponding values for BAMNet [47]. Compared with GLIA-Net [33], the average sensitivity and precision of DCAU-Net were higher by 6.86% and 2.66%, respectively. This demonstrates the effectiveness of the currently proposed method. It can be seen from the prediction maps (Fig. 8) for different models on the test set that the model detected the only lesion area in the background, and segmented the lesion more accurately.

**Table 4:** Comparison of the proposed model with other attention modules

Model	Dice	Sensitivity	Specificity	Precision
SENet [45]	0.6849	0.6206	0.9995	0.6764
SKNet [46]	0.6756	0.6172	0.9995	0.6915
BAMNet [47]	0.7451	0.7099	0.9997	0.7593
Ours	0.7455	0.7881	0.9996	0.7881

**Table 5:** Comparison of the performances of some state-of-the-art models on the ADAM test set

Model	Dice	Sensitivity	Specificity	Precision
MIP + 2D CNN	0.6642	0.7201	0.9993	0.5865
HeadXNet [29]	0.6462	0.7473	0.9994	0.6771
DeepMedic [32]	0.7421	0.7247	0.9997	0.7521
GLIA-Net [33]	0.7443	0.7195	0.9997	0.7615
DAResUNet [30]	0.7376	0.7014	0.9995	0.7553
3D U-Net [37]	0.6544	0.7181	0.9993	0.6036
Ours	0.7455	0.7881	0.9996	0.7881

**Fig. 9:**
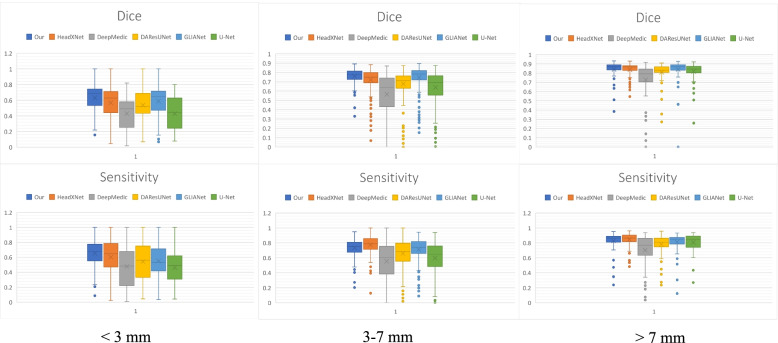
Box plots of Dice metric values and sensitivities, for different models, with respect to segmenting images with three sizes of intracranial aneurysms

The universality of DCAU-Net was further tested by using it on the clinical dataset provided by the Affiliated Hospital of Qingdao University. Comparative experiments were performed using the modeling approach proposed in the literature. These networks used the same preprocessing method for ensuring quantification. After ten-fold cross-validation, the final test results were obtained, and are listed in Table 6. Figure 9 shows a box plot, which describes the quantitative segmentation results (the Dice metric and sensitivity values) for the test set obtained after tenfold cross-validation using different methods. Table 7 lists the number of parameters for the six networks, the average duration of a training round, and the average test time for a single 3D TOF MRA-acquired image.

**Table 6:** Comparison of the segmentation performances of different methods, on the dataset of the Affiliated Hospital of Qingdao University

Model	< 3 mm			3–7 mm			> 7 mm		
	Dice	Sensitivity	Specificity	Dice	Sensitivity	Specificity	Dice	Sensitivity	Specificity
HeadXNet [29]	0.5680	0.6029	0.9998	0.7207	0.7746	0.9996	0.8383	0.8449	0.9983
DeepMedic [32]	0.4296	0.4759	0.9897	0.5655	0.5541	0.9990	0.7280	0.7028	0.9938
GLIA-Net [33]	0.4015	0.4707	0.9993	0.7028	0.7059	0.9996	0.8253	0.7899	0.9986
DAResUNet [30]	0.5369	0.5412	0.9998	0.6791	0.8265	0.6584	0.8784	0.8929	0.9979
U-Net [36]	0.4286	0.4610	0.9995	0.6393	0.6585	0.9951	0.8191	0.8069	0.9975
Ours	0.6303	0.7103	0.9998	0.7607	0.7338	0.9997	0.8412	0.8267	0.9986

**Table 7:** Comparison of computational time and memory metrics, for the dataset of aneurysms supplied by the Affiliated Hospital of Qingdao University

Method	Parameters	Train time (s)	Test time (s)		
			< 3 mm	3–7 mm	> 7 mm
HeadXNet [29]	101.59 M	423.67	2.01	1.85	1.92
DeepMedic [32]	2.51 M	396.14	1.41	1.25	1.33
GLIA-Net [33]	49.95 M	379.31	2.09	1.86	1.92
DAResUNet [30]	71.97 M	425.85	1.52	1.54	1.51
U-Net [36]	5.01 M	367.04	1.41	1.37	1.41
Ours	38.35 M	400.74	1.38	1.26	1.36

As shown in Table 6, the DCAU-Net network exhibited the highest average Dice value on the segmentation of three different-size aneurysms; the average sensitivity was higher than that for most of the other models. HeadXNet [29] adopts a 50-layer SE-ResNet as the encoder part, while the decoder part uses a transposed convolution. It can segment aneurysms more accurately than the currently proposed method, but its segmentation of the aneurysm edge regions is relatively rough. DeepMedic [32] is a dual-path network that accepts two different-size aneurysm images separately, and then unifies the feature map sizes of the two paths through up-sampling, for feature fusion. GLIA-Net [33] combines the global location information network and a local segmentation network for ensuring that both global and local information of the image can be captured. It poorly segments aneurysms with large size variations, especially small lesions. DAResUNet [30] is a U-Net network based on residual blocks, which introduces dual attention modules into the bottom layer of the network, for learning long-range context feature information. This model only considers expanding the learning of semantic information at the bottom layer and ignores the extraction of detailed information from the shallow network. U-Net [36] uses special software to create large-size markers instead of manual markers, for improving the accuracy with respect to the ground truth data, which ensures that the feature information obtained during the network training is more accurate. However, the segmentation performance of this model on clinical data is sub-optimal. The currently proposed model uses the CBAM in the feature extraction step, focusing on non-salient feature regions. This ensures that the aneurysm contextual information is learned in the largest range, and fuses the pixel sequence information of different scales during up-sampling. Both semantic information and edge information are effectively learned, and the method’s segmentation performance on intracranial aneurysms with large size differences is improved. According to Table 7, the algorithm parameters of the currently proposed method take up 38.35 MB. Although this implies that the memory cost of the proposed method is higher than those of the DeepMedic and U-Net models, the segmentation accuracy of intracranial aneurysms is significantly higher for the current method than for the latter models. The parameter costs of the other three comparison models are much higher than those of the currently proposed model, and the average test time for a single case of three image sizes is also higher than for the currently proposed model. It can be seen that, compared with the GLIA-Net model with similar performance, DCAU-Net requires less memory, has a lower computational time cost, and has a higher segmentation accuracy.

**Fig. 10:**
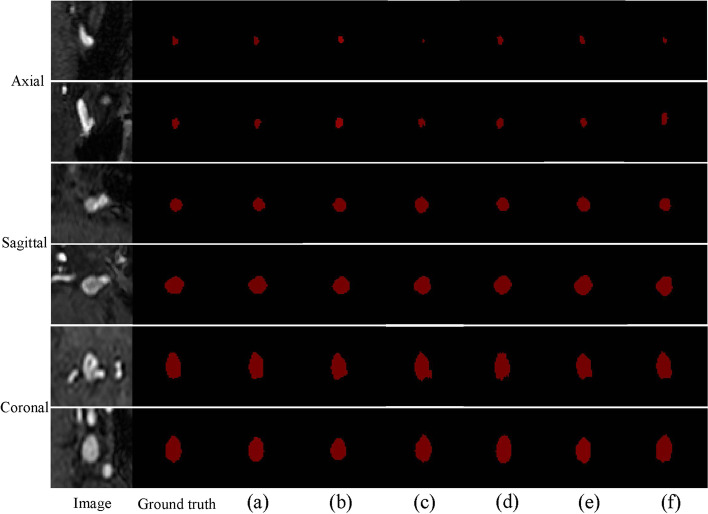
Segmentation prediction maps of the comparison model, on an example image from the test set supplied by the Affiliated Hospital of Qingdao University. **a **The currently proposed method; **b** HeadXNet; **c** DeepMedic; **d** GLIANet; **e** DAResUNet; **f** U-Net

To demonstrate the segmentation performance for individual dimensions more comprehensively, Fig. 10 shows the axial plane views for aneurysms less than 3 mm in size, the sagittal plane views for aneurysms in the 3–7 mm range, and the coronal plane views for aneurysms larger than 7 mm. Overall, both the currently proposed model and the comparison model appear to segment well larger aneurysm-containing regions with obvious texture features. Focusing on local details, the segmentation method proposed in this study is more accurate, yielding results that are closer to those obtained using the manual segmentation mask. The segmentation performance of DCAU-Net is better for smaller aneurysms (< 3 mm).

The model in this study verified the effectiveness of each component on the MICCAI 2020 ADAM dataset. Compared with other models, this network was robust. It extracted more contextual information with respect to segmenting different-size aneurysms, and exhibited a higher segmentation accuracy. It can more accurately segment the edges of intracranial aneurysms, and is likely to provide efficient guidance for medical diagnosis of aneurysm-containing areas. The memory demands are higher, but the test time is shorter, and the segmentation performance is better. This provides a more valuable reference to clinicians for diagnosing aneurysm-containing regions, which not only reduces their workload but also results in more accurate diagnostics.

Although the model in this study demonstrated good segmentation performance on different-size aneurysms, some challenges remain. In the test phase, a local image input network was used instead of inputting the entire original image. Therefore, we will specifically study the entire aneurysm image input model in the next step, improving the model’s robustness and increasing the accuracy of the aneurysms’ segmentation. The present model only considered experiments on MRA-acquired aneurysm-containing images. The accuracy of the method with respect to the imaging data acquired using other imaging techniques (such as CTA and DSA) is unknown, and additional validation is needed for demonstrating the model’s better generalizability. This will be the focus of future studies.

## Conclusions

This paper proposed a method for segmenting 3D TOF MRA-acquired images, for detection of intracranial aneurysms, using deep learning-based DCAU-Net. The proposed algorithm differs from conventional algorithms, in that it does not rely on manual feature extraction and parameter selection, and enables real-time segmentation of aneurysm-containing regions. The proposed network consists of three structures: (1) an encoder, (2) a decoder, and (3) multiscale fusion. Dense connections enhance the information transfer between the network’s layers, alleviating the problem of the vanishing gradient during the model’s training, multiplexing a large number of target features, and reducing the training cost to a certain extent. While reducing the number of parameters, dense connections allow to extract as many aneurysm-related features of different sizes and shapes as possible. The CBAM is added after dense connections. The CAM performs feature allocation according to the input and learns the feature information of different channels, while the SAM effectively focuses on the spatial information of the target region of the aneurysm, for improving the segmentation accuracy. The combination of the two sub-modules highlights the features of artificially marked areas, which is conducive to the segmentation of images containing intracranial aneurysm regions. In addition, this study fuses the feature maps during upsampling to obtain rich semantic information about the deep network and the detailed information about the shallow network at the same time, further improving the segmentation performance of the method with respect to aneurysm-containing images.

DCAU-Net performed better than other advanced models on segmentation of images containing different-size aneurysms, and has clinical utility. This network is likely to be helpful for recognizing small-scale lesions in medical images. It can assist doctors in diagnosing tumor areas, speeding up the work process, facilitating the timely detection of small-size tumors, slowing down disease-related deterioration, and prompting medical professionals to formulate more sensible and reasonable diagnosis and treatment plans.

## Data Availability

Data related to the current study are available from the corresponding author upon reasonable request.

## Abbreviations

SAH: Subarachnoid hemorrhage
MRA: Magnetic resonance angiography
3D: Three-dimensional
FCN: Fully convolutional network
TOF: Time of flight
CNN: Convolutional neural network
CTA: Computed tomography angiography
MIP: Maximal intensity projection
2D: Two-dimensional
DSA: Digital subtraction angiography
CBAM: Convolutional block attention module
MFB: Multiscale fusion block
GN: Group normalization
BN: Batch normalization
RReLU: Randomly rectified linear unit
MLP: Multilayer perceptron
ReLU: Rectified linear unit
CAM: Channel attention module
SAM: Spatial attention module

## Code availability

Some of the codes generated or used during the study are available from the corresponding author upon request.
