# Correction: DCAU-Net: dense convolutional attention U-Net for segmentation of intracranial aneurysm images

**DOI:** 10.1186/s42492-022-00110-7

**Published:** 2022-05-08

**Authors:** Wenwen Yuan, Yanjun Peng, Yanfei Guo, Yande Ren, Qianwen Xue

**Affiliations:** 1 College of Computer Science and Engineering, Shandong University of Science and Technology, Qingdao, 266590 China; 2 The Department of Radiology, the Afliated Hospital of Qingdao University, Qingdao, 266000 China; 3 Qingdao Maternal & Child Health and Family Planning Service Center, Qingdao, 266034 China

**Correction to: Vis Comput Ind Biomed Art 5, 9 (2022)**

**https://doi.org/10.1186/s42492-022-00105-4**

Following publication of the original article [1], the authors identified an error in Figs. 6 and 8 due to a typesetting error. The correct figures are given below.

**Fig. 6:**
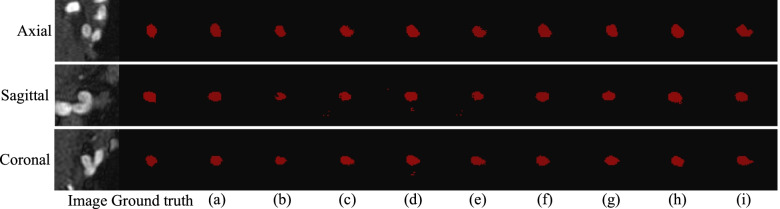
Prediction maps for the models with different components, on the MICCAI 2020 ADAM testing set. The first column is the MRA image of an aneurysm, while the second column shows the manually obtained marking mask. **a** The proposed model; **b** U-Net; **c** U-Net with the dense block; **d** U-Net + CAM; **e** U-Net + SAM; **f** U-Net + CBAM; **g** U-Net + dense block + CBAM; **h** BN only; **i** With ReLU activation

**Fig 8:**
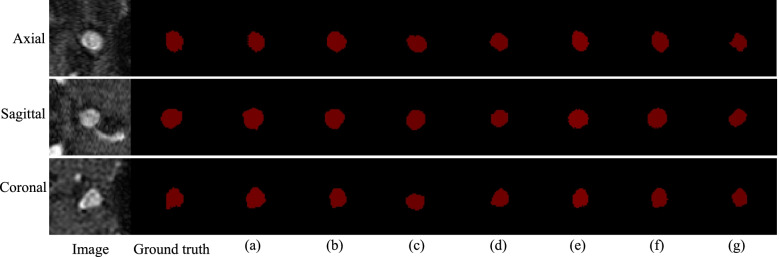
Prediction maps of different models, on the ADAM test set. **a** The proposed model; **b** MIP + 2D CNN; **c** HeadXNet; **d** DeepMedic; **e** GLIANet; **f** DAResUNet; **g** 3D U-Net

In addition, the authors also identified some errors in the sections “*The CBAM*” and “*Results and discussion*” due to negligence. The correct information is given below:

“where σ represents the sigmoid function. Note that the MLP weights, W0 and W1, are shared for both inputs, and the rectifed linear unit (ReLU) activation function is followed by *W*_0_.”

“The results of these experiments show that the Dice value for DCAU-Net reached 74.55% on the testing set. The basic U-Net network exhibited the lowest Dice of 46.29%, which is not suitable for segmenting aneurysm-containing images. The Dice value for U-Net with dense blocks was 7.95% higher compared with that for U-Net alone, proving the effectiveness of dense connections. The combination of U-Net and CBAM was less sensitive than the system that used only the SAM, but the Dice value was higher than that for U-Net combined with a single spatial or channel attention, indicating the effectiveness of the CBAM for segmenting aneurysm-containing regions. To validate the effectiveness of the MFB, a multi-scale fusion block was added to the improved U-Net up-sampling part. The Dice value increased by 1.63%, and the sensitivity increased by 5.03%, indicating that the MFB effectively improved the segmentation performance on aneurysm-containing images. The Dice value and the sensitivity of the traditional convolution block using the BN layer were, respectively, 8.43% and 10.52% lower than those of the currently proposed algorithm. This shows that the GN layer effectively improved the segmentation accuracy for aneurysm-containing images while reducing the memory space. The Dice value obtained using the RReLU function was 17.24% higher than that obtained using the ReLU function, proving the effectiveness of the RReLU function.”

The original article [1] has been corrected.
